# Hematological inflammatory indices and their relationship to the risk of hypertension

**DOI:** 10.4178/epih.e2026008

**Published:** 2026-02-04

**Authors:** Ju Young Jung, Chang-Mo Oh, Jae-Hong Ryoo, Sung Keun Park

**Affiliations:** 1 Total Healthcare Center, Kangbuk Samsung Hospital, Sungkyunkwan University School of Medicine, Seoul, Korea; 2 Departments of Preventive Medicine, Kyung Hee University School of Medicine, Seoul, Korea; 3 Departments of Occupational and Environmental Medicine, Kyung Hee University School of Medicine, Seoul, Korea

**Keywords:** Inflammation, Hypertension, Blood cells

## Abstract

**OBJECTIVES:**

Chronic inflammation has been implicated in the development of hypertension in numerous previous studies. However, evidence regarding the association between hematological inflammatory indices derived from complete blood count tests and the long-term risk of hypertension remains limited. Therefore, this study aimed to investigate the association between various hematological inflammatory indices and the risk of incident hypertension in a large cohort study.

**METHODS:**

We analyzed data from a large Korean cohort (n=128,241). The incident risk of hypertension was evaluated according to quartiles of the neutrophil-to-lymphocyte ratio (NLR), monocyte-to-lymphocyte ratio (MLR), platelet-to-lymphocyte ratio (PLR), systemic immune-inflammation index (SII), and systemic inflammatory response index (SIRI) using Cox proportional hazards models. Additional analyses were conducted for high-sensitivity C-reactive protein (hsCRP) levels and stratified by gender.

**RESULTS:**

During a median follow-up of 6.8 years, 18,503 participants (14.4%) developed hypertension. Higher quartiles of SII, SIRI, and NLR were significantly associated with an increased risk of incident hypertension in both genders. PLR showed a clearer positive association in women, whereas MLR demonstrated only marginal associations. These patterns were consistent with the associations observed for hsCRP.

**CONCLUSIONS:**

These findings suggest that elevated hematological inflammatory indices above certain thresholds are associated with an increased risk of hypertension, even among young and generally healthy individuals.

## GRAPHICAL ABSTRACT

[Fig f2-epih-48-e2026008]

## Key Message

• Hematological inflammatory indices (SII, SIRI, NLR, MLR, and PLR) above specific values were associated with the increased risk of incident hypertension.

• SII, SIRI, and NLR showed dose–response relationships with the risk of hypertension in both men and women.

• Hematological inflammatory indices may serve as accessible markers for the risk stratification of hypertension.

## INTRODUCTION

The prevalence of hypertension is rapidly increasing worldwide. Despite substantial public health investments in hypertension prevention, the number of individuals aged 30–79 years with hypertension doubled between 1990 and 2019 [1]. Currently, more than 1.3 billion adults are affected by hypertension, with most remaining undiagnosed or failing to achieve target blood pressure (BP) levels, particularly in middle-income to low-income countries with limited healthcare resources [2-4]. Consequently, hypertension is responsible for more than 10 million deaths globally each year [4]. To address this growing public health burden, there is an increasing need for cost-effective and easily accessible tools to identify individuals at high risk of hypertension prior to its onset. In this context, hematological inflammatory indices have recently gained attention as potential biomarkers for early hypertension.

Recent studies have suggested that hematological inflammatory indices may be associated with an increased risk of hypertension [5-7]. A meta-analysis reported that individuals with hypertension have higher neutrophil-to-lymphocyte ratios (NLRs) than those with normal BP [5]. Another study demonstrated that individuals with non-dipper hypertension were 2.11 times more likely to exhibit higher platelet-to-lymphocyte ratios (PLRs) than those with dipper hypertension [6]. A Chinese cohort study further showed that the systemic inflammatory response index (SIRI) and the systemic immune-inflammation index (SII) were independently associated with an increased risk of hypertension [7]. Collectively, these findings suggest that hematological inflammatory indices may serve as markers linking chronic systemic inflammation to the development of hypertension. However, the existing evidence remains insufficient to establish a consistent positive association between hematological inflammatory indices and hypertension [8-10]. Several epidemiological studies have reported U-shaped [8] or non-linear relationships [9] between these indices and hypertension. One study also demonstrated that the association between NLR and the risk of hypertension was no longer significant after adjustment for body mass index (BMI) [10]. In addition, few studies have longitudinally evaluated multiple hematological inflammatory indices in relation to the risk of incident hypertension using comparative analyses.

Therefore, this study aimed to evaluate the risk of incident hypertension in relation to levels of commonly used hematological inflammatory indices. To achieve this objective, we conducted a large-scale retrospective cohort study using data from a comprehensive health check-up program in a large Korean adult population.

## MATERIALS AND METHODS

### Study participants and exclusion

We obtained hematological, biochemical, and other relevant data from the Kangbuk Samsung Health Study. All participants underwent annual or biennial health check-ups at Kangbuk Samsung Hospital in accordance with Korea’s Industrial Safety and Health Law, which mandates regular health examinations for employees. The costs of these examinations were covered by the company, and both employees and their family members were eligible to receive company-supported health check-ups.

**Figure 1.**
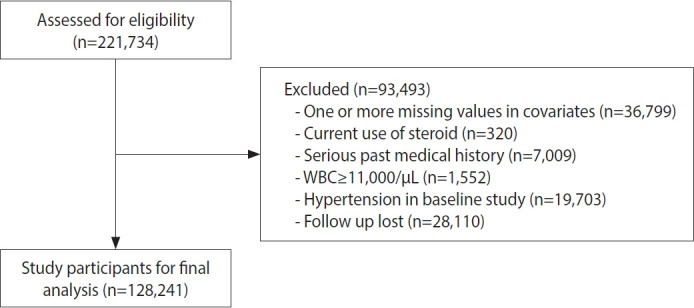
Flow chart of enrolled study participants.

Between March 2011 and December 2012, a total of 221,954 individuals underwent health check-ups, and we initially enrolled 221,734 participants with complete blood count (CBC) data. Among these individuals, 36,799 were excluded due to missing values in covariates such as triglycerides, alcohol intake, or body weight. We further excluded 320 participants who were current steroid users and 7,009 participants with a serious past medical history (e.g., cancer, coronary heart disease, or chronic obstructive pulmonary disease) that could influence systemic inflammation or CBC results. In addition, 1,552 participants with leukocytosis (white blood cell count ≥11,000/μL) were excluded. Among the remaining 176,054 participants, we excluded 19,311 individuals who had hypertension at baseline and 28,502 individuals who did not have follow-up visits between January 2013 and December 2019. Finally, 128,241 participants were included in the final analysis (Figure 1).

### Clinical/biochemical data and hematological inflammatory indices

All participants completed a detailed questionnaire assessing health-related behaviors, medical history, and medication use before the health check-up. The questionnaire on alcohol consumption assessed the type of alcoholic beverage (e.g., beer) as well as the frequency and amount of alcohol consumed for each beverage type. Based on these responses, alcohol intake was calculated and expressed as daily alcohol consumption (g/day). Smoking status was categorized into 4 groups: never smoker, former smoker, current smoker, and no response. Physical activity was assessed using the Korean-validated short form of the International Physical Activity Questionnaire (IPAQ), and participants were classified into 3 categories according to IPAQ guidelines: low, moderate, and high physical activity levels (http://www.ipaq.ki.se).

After completion of the questionnaire, anthropometric measurements and laboratory tests, including CBC and other biochemical analyses, were performed for all participants. Body weight and height were measured automatically, and BMI was calculated as weight (kg) divided by height squared (m²). Hypertension was defined by any of the following criteria: a prior diagnosis, current use of antihypertensive medication, or measured systolic blood pressure (SBP) ≥140 mmHg or diastolic blood pressure (DBP) ≥90 mmHg at baseline or during follow-up. These hypertension thresholds were based on the Korean Hypertension Society guidelines [11]. If hypertension was first identified during follow-up, that time point was considered the onset of hypertension, and follow-up was terminated. Participants who did not develop hypertension by the end of follow-up were considered free of incident hypertension. Diabetes mellitus was defined by any of the following criteria: fasting glucose ≥126 mg/dL, hemoglobin A1c (HbA1c) ≥6.5%, current use of antidiabetic medication, or a prior diagnosis of diabetes [12]. Blood samples were collected from the antecubital vein after fasting for more than 12 hours. Fasting serum glucose was measured using the hexokinase method, and HbA1c was assessed using an immunoturbidimetric assay with a Cobas Integra 800 automatic analyzer (Roche Diagnostics, Basel, Switzerland). Insulin concentrations were measured using immunoradiometric assays (Biosource, Nivelles, Belgium). The homeostasis model assessment of insulin resistance (HOMA-IR) was calculated using the following formula: HOMA-IR=fasting serum insulin (μU/mL)×fasting serum glucose (mg/dL)/405. CBC testing was performed using flow cytometry (XE-2100 Hematology Automated Analyzer; Sysmex, Kobe, Japan). Total cholesterol and triglyceride concentrations were measured enzymatically, while low-density lipoprotein (LDL) cholesterol was measured using homogeneous enzymatic colorimetric assays and selective inhibition methods, respectively (Advia 1650 Autoanalyzer; Bayer Diagnostics, Leverkusen, Germany). High-sensitivity C-reactive protein (hsCRP) was measured using immunoturbidimetry with a Modular DPP analyzer (Roche Diagnostics, Tokyo, Japan). Hematological inflammatory indices were calculated using formulas reported in previous studies [13-17].

• Systemic immune-inflammation index (SII): (N×P)/L

• Systemic inflammatory response index (SIRI): (N×M)/L

• Neutrophil-to-lymphocyte ratio (NLR): N/L

• Monocyte-to-lymphocyte ratio (MLR): M/L

• Platelet-to-lymphocyte ratio (PLR): P/L

(N, P, M, and L denote neutrophil, platelet, monocyte, and lymphocyte counts, respectively)

### Statistical analysis

Baseline clinical, biochemical, and hematological inflammatory data are presented as means±standard deviations for continuous variables and as proportions for categorical variables. Given known gender-related differences in the prevalence of hypertension and systemic inflammation, subgroup analyses stratified by gender were conducted (p for interaction: p<0.001 for SII, p=0.104 for SIRI, p=0.008 for NLR, p=0.334 for MLR, and p<0.001 for PLR). Baseline characteristics of men and women were compared using independent *t*-tests for continuous variables and chi-square tests for categorical variables. To address the potential for statistically significant differences arising from large sample sizes, Cohen’s *d* was also calculated as a standardized mean difference, with values of 0.2, 0.5, and 0.8 representing small, medium, and large effect sizes, respectively.

Cox proportional hazards models were used to estimate unadjusted and multivariable-adjusted hazard ratios (HRs) with 95% confidence intervals (CIs) for incident hypertension across quartiles of systemic inflammatory indices (SII, SIRI, NLR, MLR, and PLR). The lowest quartile of each hematological inflammatory index served as the reference group. Covariates included in multivariable models were selected based on known associations with both hypertension and systemic inflammation and included age, gender, diabetes mellitus, physical activity, alcohol intake, smoking status, BMI, LDL cholesterol, triglycerides, SBP, and HOMA-IR (gender was excluded from gender-specific models). For each quartile, the number of incident cases, incidence density per 1,000 person-years, and total person-years of follow-up were calculated. Linear trends were evaluated using the median value of each quartile. The proportional hazards assumption was assessed using log–log survival plots. Relative risks (RRs) with 95% CIs were also calculated for hypertension across quartiles.

Multicollinearity among covariates was assessed using variance inflation factors (VIFs), confirming that none exceeded 10. A separate analysis using hsCRP quartiles was conducted to enable comparison with hematological inflammatory indices. Because of the substantial proportion of missing hsCRP values (n=17,176), results from this analysis are presented in a separate table. Kaplan–Meier curve analysis was performed to evaluate hypertension-free survival according to quartiles of the 5 hematological inflammatory indices.

All statistical analyses were conducted using R version 4.5.0 (R Foundation for Statistical Computing, Vienna, Austria). A 2-sided p-value of less than 0.05 was considered statistically significant.

### Ethics statement

Ethical approval for the study protocol and data analysis was obtained from the Institutional Review Board (IRB) of Kangbuk Samsung Hospital (IRB No. KBSMC 2022-08-041). The IRB approved a waiver of informed consent because the study involved retrospective analysis of de-identified data obtained from routine health check-ups.

## RESULTS

Table 1 presents a summary of the baseline clinical, biochemical, hematological, and inflammatory characteristics of the study population, stratified by gender. The cohort consisted of young, relatively healthy individuals, with a mean age of 38.4±6.5 years, and men were slightly older than women (38.9 vs. 37.8 years; p<0.001; Cohen’s *d*=−0.18). Men exhibited a higher burden of cardiometabolic risk factors, including a significantly greater prevalence of diabetes mellitus and current smoking, as well as elevated BMI, LDL cholesterol, triglycerides, and higher daily alcohol intake, with moderate to large effect sizes (Cohen’s *d* ranging from −0.61 to −1.88). Notably, men also had significantly higher SBP (113.0 vs. 101.7 mmHg; p<0.001; Cohen’s *d*=−1.11) and DBP (72.2 vs. 64.7 mmHg; p<0.001; Cohen’s *d*=−0.96), indicating pronounced gender differences in BP levels with large effect sizes. In contrast, women had higher platelet counts and elevated hematological inflammatory indices, including SII, NLR, and PLR (p<0.001; Cohen’s *d*=0.18–0.57), suggesting a more active inflammatory profile despite more favorable overall metabolic health. However, men showed higher SIRI and MLR values (Cohen’s *d*=−0.11 and −0.13, respectively), indicating distinct patterns of immune activation. Over a median follow-up of 6.8 years, 18,503 participants (14.4%) developed incident hypertension, with a significantly higher incidence in men (20.6%) than in women (5.8%) (p<0.001; Cohen’s *d*=−0.80), reflecting a large effect size and highlighting a robust gender-based disparity in hypertension risk.

Table 2 presents the unadjusted and multivariable-adjusted HRs and RRs with 95% CIs for hypertension across quartiles of SII, SIRI, NLR, MLR, and PLR in the overall study population. In multivariable-adjusted analyses, higher quartiles of SII (Q2: 1.05 [1.01 to 1.10]; Q3: 1.14 [1.10 to 1.19]; Q4: 1.23 [1.18 to 1.28]; p for trend <0.001), SIRI (Q3: 1.10 [1.05 to 1.15]; Q4: 1.16 [1.11 to 1.21]; p for trend<0.001), NLR (Q2: 1.10 [1.06 to 1.15]; Q3: 1.09 [1.05 to 1.14]; Q4: 1.19 [1.14 to 1.24]; p for trend<0.001), and MLR (Q2: 1.05 [1.01 to 1.10]; Q3: 1.07 [1.03 to 1.12]; Q4: 1.11 [1.07 to 1.16]; p for trend<0.001) were associated with an increased risk of hypertension compared with the lowest quartile (Q1; reference). In contrast, PLR demonstrated a decreased unadjusted HR but an increased multivariable-adjusted hazard ratio (aHR), with a significant association observed in the highest quartile (Q4: aHR, 1.21; 95% CI, 1.16 to 1.26; p for trend<0.001). These apparently contradictory findings were largely attenuated in gender-specific subgroup analyses, reflecting the combination of higher PLR levels and lower hypertension incidence among women. Results from gender-stratified analyses are presented in Tables 3 and 4. Overall, both men and women demonstrated similar dose–response relationships for SII, SIRI, NLR, and MLR, with women generally exhibiting higher multivariable-aHRs. Among men, PLR showed no significant association in unadjusted analyses but was associated with an increased risk of hypertension in the highest quartile after multivariable adjustment (Q4: aHR, 1.13; 95% CI, 1.08 to 1.18). In contrast, women demonstrated significant associations for PLR in both unadjusted and adjusted analyses, with progressively higher risk across quartiles (Q3: aHR, 1.15; 95% CI, 1.04 to 1.28; Q4: aHR, 1.37; 95% CI, 1.24 to 1.51; p for trend<0.001). Although RR risk estimates tended to be slightly lower in magnitude, RRs exhibited patterns comparable to those observed for HRs.

Table 5 presents the association between hsCRP quartiles and the risk of incident hypertension in all participants and in gender-specific analyses. In unadjusted models, a strong positive association was observed across increasing hsCRP quartiles (Q2: 1.66 [1.58 to 1.74], Q3: 2.12 [2.02 to 2.22]; Q4: 2.50 [2.39 to 2.62]; p for trend <0.001). After adjustment for potential confounding variables, these associations were attenuated but remained statistically significant (Q2: aHR, 1.07; 95% CI, 1.02 to 1.13; Q3: aHR, 1.10; 95% CI, 1.04 to 1.16; Q4: aHR, 1.17; 95% CI, 1.11 to 1.24; p for trend <0.001). In gender-stratified analyses, similar patterns were observed in both men and women, although women generally exhibited higher unadjusted HRs and aHRs.

Kaplan–Meier curves illustrating hypertension-free survival across quartiles of SII, SIRI, NLR, MLR, and PLR are presented in Supplementary Materials 1-3 (all participants), 2 (men), and 3 (women). Across all analyses, Kaplan–Meier curves demonstrated patterns consistent with the unadjusted HRs observed for each quartile group.

## DISCUSSION

In this study, we demonstrated a positive association between chronic inflammation, as assessed by hematological inflammatory indices, and the risk of developing hypertension. Despite the relatively small magnitude of the associations (HRs <1.5), SII, SIRI, and NLR exhibited clear dose–response relationships with hypertension risk. In addition, MLR and PLR levels above specific quartiles were significantly associated with a marginally increased risk of incident hypertension. Taken together, these findings suggest that several hematological inflammatory indices may help identify individuals at elevated risk of hypertension, even among young and apparently healthy adults.

Our findings are consistent with previous studies reporting significant associations between elevated hematological inflammatory indices and an increased risk of hypertension. For example, NLR has been recognized as a reliable marker of systemic inflammation across a range of conditions, including malignancy [18], cardiovascular disease [13], sepsis, and pneumonia [19], and has also been linked to incident hypertension [5,20]. A large cohort study of 28,850 Chinese adults reported that individuals in the highest NLR quintile had a significantly higher risk of hypertension (HR, 1.23; 95% CI, 1.06 to 1.43) [20]. Similar associations have been observed for other hematological inflammatory indices. A longitudinal cohort study with a 6-year follow-up confirmed that both SII and SIRI were independently associated with an increased long-term risk of hypertension [21]. In line with these prior findings, our results further support the potential association between elevated hematological inflammatory indices and an increased risk of incident hypertension.

The hematological inflammatory indices examined in this study (SII, SIRI, NLR, MLR, and PLR) have traditionally been regarded as markers reflecting neutrophilia and relative lymphopenia in acute inflammatory states. However, accumulating evidence suggests that these indices also reflect chronic inflammatory responses and may serve as predictors of cardiovascular diseases closely related to hypertension. NLR is closely linked to the role of chronic inflammation and oxidative stress in the pathophysiology of atherosclerosis and endothelial dysfunction, which may explain its association with cardiovascular events and mortality [22]. Buonacera et al. [23] reported that NLR is a simple and inexpensive biomarker reflecting the balance between acute and chronic inflammation and adaptive immunity. PLR may serve as a promising indicator of inflammation and vascular dysfunction in metabolic syndrome [24]. MLR reflects monocyte-driven chronic inflammatory pathways and has been associated with adverse cardiovascular outcomes [25]. In addition, SII has emerged as a potential marker of cardiovascular disease, reflecting chronic inflammatory burden [26]. Chronic inflammation is associated with oxidative stress, endothelial dysfunction, and cytokine secretion, which represent plausible mechanisms linking these indices to the development of hypertension. Nevertheless, we acknowledge that our study cannot establish precise underlying mechanisms because of the inherent limitations of observational research. Accordingly, further studies are warranted to clarify the causal pathways linking hematological inflammatory indices to hypertension development.

Our results also suggest the presence of gender-specific differences in the association between hematological inflammatory indices and hypertension risk. Although the incidence of hypertension was higher in men, women exhibited higher hazard ratios across indices. Several factors may contribute to this observation, including gender-related differences in hypertension risk factors and transitions to menopause. Compared with women, men displayed more unfavorable baseline clinical characteristics, including poorer lifestyle factors such as higher smoking prevalence, higher BMI, elevated SBP, increased HOMA-IR, and higher levels of LDL cholesterol and triglycerides. These adverse baseline conditions may partly explain the higher incidence of hypertension observed among men. Consequently, the influence of systemic inflammation on hypertension risk may appear relatively attenuated in men compared with women. In addition, a proportion of women participants likely transitioned to menopause during the 6.8-year follow-up period. Menopause is associated with a substantial increase in hypertension risk, partly due to attenuation of estrogen-mediated immune protection [27]. Clinically, postmenopausal women exhibit increased expression of inflammatory markers, including monocyte chemoattractant protein-1, tumor necrosis factor-α, interferon-γ, and interleukin-6, as well as marked shifts in the balance between pro-inflammatory and anti-inflammatory immune cell populations [28,29]. The postmenopausal state is therefore more strongly predisposed to hypertension through an enhanced pro-inflammatory immune milieu. Accordingly, women with higher baseline inflammatory index levels may have been particularly vulnerable to immune-mediated hypertension, which may explain the stronger observed associations.

Several limitations of the present study should be acknowledged. First, most participants were relatively young and healthy; therefore, levels of chronic systemic inflammation were likely lower than those in the general population, potentially underestimating associations for some hematological inflammatory indices. Second, hematological inflammatory indices were calculated using a single baseline CBC measurement. White blood cell composition and counts can vary according to factors such as season [30] and circadian rhythms [31], which may have introduced residual confounding. Third, although the HRs for hypertension associated with hematological inflammatory indices were statistically significant, their magnitudes were modest (all <1.5). In large-scale studies, statistical significance does not necessarily imply clinical relevance. Accordingly, our findings should be interpreted as demonstrating a positive association between hematological inflammatory indices and hypertension risk rather than as evidence of their predictive utility for incident hypertension.

In conclusion, our study suggests that increases in SII, SIRI, NLR, and PLR above specific levels are significantly associated with an increased risk of incident hypertension. These associations were observed in both men and women, with a slightly stronger magnitude noted in women. Overall, our findings provide additional evidence supporting previous studies that have reported positive associations between elevated hematological inflammatory indices and hypertension.

## Figures and Tables

**Figure f2-epih-48-e2026008:**
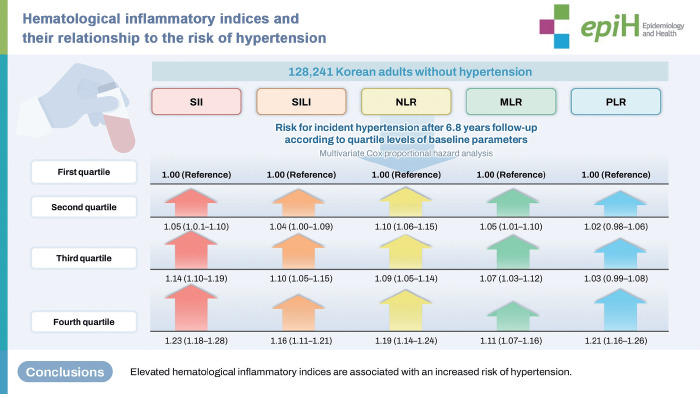


**Table 1.** Baseline clinical characteristics of study participants

Characteristics	All participants	Women	Men	p-value	Cohen’s *d* (95% CI)^1^
Total (n)	128,241	53,093	75,148		
Age (yr)	38.4±6.5	37.8±6.2	38.9±6.7	<0.001	–0.18 (–0.19, –0.16)
Triglyceride (mg/dL)	110.7±74.2	80.3±43.4	132.3±83.3	<0.001	–0.75 (–0.76, –0.74)
LDL-cholesterol (mg/dL)	119.9±31.4	108.5±28.5	127.9±30.9	<0.001	–0.65 (–0.66, –0.64)
Systolic blood pressure (mmHg)	108.4±11.6	101.7±10.5	113.0±10.0	<0.001	–1.11 (–1.12, –1.10)
Diastolic blood pressure (mmHg)	69.1±8.6	64.7±7.7	72.2±7.9	<0.001	–0.96 (–0.97, –0.95)
BMI (kg/m²)	23.1±3.1	21.4±2.8	24.2±2.8	<0.001	–0.99 (–1.00, –0.98)
Average alcohol use (g/day)	14.0±21.1	5.9±11.8	19.8±24.2	<0.001	–0.69 (–0.71, –0.68)
Current smoker	29,359 (22.9)	1,041 (2.0)	28,318 (37.7)	<0.001	–1.88 (–1.91, –1.84)
High physical activity	21,518 (16.8)	8,660 (16.3)	12,858 (17.1)	<0.001	–0.03 (–0.05, –0.02)
Diabetes mellitus	3,205 (2.5)	615 (1.2)	2,590 (3.4)	<0.001	–0.61 (–0.66, –0.57)
HOMA-IR	1.3±1.0	1.2±0.9	1.4±1.0	<0.001	–0.23 (–0.24, –0.22)
WBC count (10³/μL)	5.9±1.4	5.7±1.4	6.1±1.4	<0.001	–0.28 (–0.29, –0.27)
Neutrophil count (10³/μL)	3.3±1.1	3.3±1.2	3.3±1.1	<0.001	–0.07 (–0.08, –0.06)
Lymphocyte count (10³/μL)	2.1±0.5	2.0±0.5	2.2±0.5	<0.001	–0.41 (–0.42, –0.39)
Monocyte count (10³/μL)	0.4±0.1	0.3±0.1	0.4±0.2	<0.001	–0.49 (–0.50, –0.47)
Platelet count (10³/μL)	240.5±49.5	249.5±53.7	234.1±45.2	<0.001	0.32 (0.31, 0.33)
SII (10⁹ cells/L)	400.7±192.9	434.1±213.2	377.0±173.3	<0.001	0.30 (0.29, 0.31)
SIRI (10⁹ cells/L)	638.1±398.8	612.3±393.4	656.4±401.5	<0.001	–0.11 (–0.12, –0.10)
NLR	1.7±0.7	1.7±0.8	1.6±0.7	<0.001	0.18 (0.17, 0.19)
MLR	0.19±0.07	0.18±0.07	0.19±0.07	<0.001	–0.13 (–0.14, –0.12)
PLR	121.5±36.9	133.4±39.9	113.1±32.0	<0.001	0.57 (0.56, 0.59)
hsCRP (mg/dL)^2^	0.10±0.03	0.08±0.03	0.11±0.03	<0.001	–0.12 (–0.13, –0.11)
n	111,065	45,902	65,163		
Incidental case of hypertension	18,503 (14.4)	3,060 (5.8)	15,443 (20.6)	<0.001	–0.80 (–0.82, –0.77)

Values are presented as mean±standard deviation for continuous variables and number (%) for categorical variables. BMI, body mass index; LDL, low density lipoprotein; HOMA-IR, homeostatic model assessment for insulin resistance; WBC, white blood cell; SII, systemic immune-inflammation index; SIRI, systemic inflammatory response index; NLR, neutrophil-to-lymphocyte ratio; MLR, monocyte-to-lymphocyte ratio; PLR, platelet-to-lymphocyte ratio; hsCRP, high-sensitivity C-reactive protein.

1 Cohen’s *d*: 0.2 (small), 0.5 (medium), 0.8 (large).

2 Missing values in hsCRP: 17,176 (13.4%) in all participants, 7,191 (13.5%) in women, 9,985 in men (13.3%).

**Table 2.** HRs, RR and their 95% CIs for hypertension according to quartiles of SII, SIRI, NLR, MLR, and PLR

Variables^1^	Quartile 1	Quartile 2	Quartile 3	Quartile 4	p for trend
SII (n)	32,061	32,060	32,060	32,060	
Unadjusted HR	1.00 (reference)	1.06 (1.01, 1.10)	1.11 (1.06, 1.15)	1.07 (1.02, 1.11)	0.003
Multivariable adjusted HR	1.00 (reference)	1.05 (1.01, 1.10)	1.14 (1.10, 1.19)	1.23 (1.18, 1.28)	<0.001
Unadjusted RR	1.00 (reference)	1.05 (1.01, 1.10)	1.09 (1.05, 1.14)	1.05 (1.01, 1.10)	0.028
Multivariable adjusted RR	1.00 (reference)	1.05 (1.01, 1.10)	1.12 (1.08, 1.17)	1.20 (1.15, 1.25)	<0.001
Case, n (%)/PY	4,445 (13.9)/194,692	4,642 (14.5)/193,239	4,803 (15.0)/192,387	4,613 (14.4)/192,254	
Incidence density	22.8	24.0	25.0	24.0	
Range of SII (median)	≤273 (224)	273–361 (316)	361–481 (413)	≥481 (593)	
SIRI (n)	32,061	32,060	32,060	32,060	
Unadjusted HR	1.00 (reference)	1.18 (1.13, 1.23)	1.29 (1.24, 1.35)	1.42 (1.36, 1.48)	<0.001
Multivariable adjusted HR	1.00 (reference)	1.04 (1.00, 1.09)	1.10 (1.05, 1.15)	1.16 (1.11, 1.21)	<0.001
Unadjusted RR	1.00 (reference)	1.17 (1.12, 1.23)	1.28 (1.22, 1.33)	1.39 (1.34, 1.45)	<0.001
Multivariable adjusted RR	1.00 (reference)	1.03 (0.99, 1.08)	1.08 (1.03, 1.12)	1.13 (1.08, 1.18)	<0.001
Case, n (%)/PY	3,865 (12.1)/195,309	4,498 (14.0)/193,590	4,859 (15.2)/192,176	5,281 (16.5)/191,498	
Incidence density	19.8	23.2	25.3	27.6	
Range of SIRI (median)	≤387 (305)	387–545 (463)	545–774 (643)	≥774 (1,001)	
NLR (n)	32,307	32,310	32,306	32,304	
Unadjusted HR	1.00 (reference)	1.08 (1.03, 1.12)	1.05 (1.01, 1.09)	1.03 (0.99, 1.08)	0.466
Multivariable adjusted HR	1.00 (reference)	1.10 (1.06, 1.15)	1.09 (1.05, 1.14)	1.19 (1.14, 1.24)	<0.001
Unadjusted RR	1.00 (reference)	1.07 (1.03, 1.11)	1.04 (1.00, 1.09)	1.02 (0.97, 1.06)	0.965
Multivariable adjusted RR	1.00 (reference)	1.09 (1.04, 1.13)	1.08 (1.04, 1.13)	1.15 (1.11, 1.20)	<0.001
Case, n (%)/PY	4,506 (14.1)/194,189	4,779 (14.9)/192,774	4,677 (14.6)/192,937	4,541 (14.2)/192,672	
Incidence density	23.2	24.8	24.2	23.6	
Range of NLR (median)	≤1.21 (1.03)	1.21–1.52 (1.37)	1.52–1.95 (1.71)	≥1.95 (2.34)	
MLR (n)	32,064	32,074	32,045	32,058	
Unadjusted HR	1.00 (reference)	1.08 (1.04, 1.13)	1.13 (1.09, 1.18)	1.15 (1.11, 1.20)	<0.001
Multivariable adjusted HR	1.00 (reference)	1.05 (1.01, 1.10)	1.07 (1.03, 1.12)	1.11 (1.07, 1.16)	<0.001
Unadjusted RR	1.00 (reference)	1.07 (1.03, 1.12)	1.13 (1.08, 1.18)	1.14 (1.09, 1.18)	<0.001
Multivariable adjusted RR	1.00 (reference)	1.03 (0.99, 1.08)	1.06 (1.02, 1.11)	1.09 (1.04, –1.13)	<0.001
Case, n (%)/PY	4,287 (13.4)/194,056	4,568 (14.2)/192,821	4,818 (15.0)/193,159	4,830 (15.1)/192,537	
Incidence density	22.1	23.7	24.9	25.1	
Range of MLR (median)	≤0.142 (0.123)	0.142–0.175 (0.158)	0.175–0.217 (0.194)	≥0.217 (0.254)	
PLR (n)	32,061	32,060	32,060	32,060	
Unadjusted HR	1.00 (reference)	0.84 (0.81, 0.88)	0.75 (0.72, 0.78)	0.71 (0.68, 0.74)	<0.001
Multivariable adjusted HR	1.00 (reference)	1.02 (0.98, 1.06)	1.03 (0.99, 1.08)	1.21 (1.16, 1.26)	<0.001
Unadjusted RR	1.00 (reference)	0.84 (0.81, 0.87)	0.75 (0.72, 0.78)	0.71 (0.68, 0.74)	<0.001
Multivariable adjusted RR	1.00 (reference)	1.01 (0.97, 1.05)	1.03 (0.99, 1.08)	1.18 (1.13, 1.23)	<0.001
Case, n (%)/PY	5,584 (17.4)/192,036	4,722 (14.7)/193,078	4,219 (13.2)/193,832	3,978 (12.4)/193,626	
Incidence density	29.1	24.5	21.8	20.5	
Range of PLR (median)	≤96 (84)	96–116 (106)	116–140 (127)	≥140 (161)	

HR, hazard ratio; RR, relative risk; CI, confidence interval; SII, systemic immune-inflammation index; SIRI, systemic inflammatory response index; NLR, neutrophil-to-lymphocyte ratio; MLR, monocyte-to-lymphocyte ratio; PLR, platelet-to-lymphocyte ratio; PY, person years.

1 Adjusted for age, gender, diabetes mellitus, physical activity, alcohol intake, smoking, body mass index, low-density lipoprotein cholesterol, triglycerides, homeostatic model assessment for insulin resistance, and systolic blood pressure.

**Table 3.** HRs, RR, and their 95% CIs for hypertension according to quartiles of SII, SIRI, NLR, MLR, and PLR in men

Variables^1^	Quartile 1	Quartile 2	Quartile 3	Quartile 4	p for trend
SII (n)	18,787	18,787	18,787	18,787	
Unadjusted HR	1.00 (reference)	1.09 (1.04, 1.15)	1.17 (1.12, 1.23)	1.25 (1.19, 1.31)	<0.001
Multivariable adjusted HR	1.00 (reference)	1.06 (1.01, 1.11)	1.11 (1.06, 1.16)	1.20 (1.14, 1.25)	<0.001
Unadjusted RR	1.00 (reference)	1.09 (1.04, 1.14)	1.16 (1.11, 1.22)	1.23 (1.17, 1.28)	<0.001
Multivariable adjusted RR	1.00 (reference)	1.06 (1.01, 1.11)	1.10 (1.05 , 1.15)	1.17 (1.11, 1.22)	<0.001
Case, n (%)/PY	3,499 (18.6)/113,493	3,770 (20.1)/111,977	3,998 (21.3)/111,404	4,176 (22.2)/110,535	
Incidence density	30.8	33.7	35.9	37.8	
Range of SII (median)	≤261 (217)	261–342 (302)	342–451 (390)	≥451 (552)	
SIRI (n)	18,787	18,787	18,787	18,787	
Unadjusted HR	1.00 (reference)	1.09 (1.04, 1.14)	1.17 (1.12, 1.23)	1.25 (1.19, 1.30)	<0.001
Multivariable adjusted HR	1.00 (reference)	1.04 (0.99, 1.09)	1.09 (1.04, 1.14)	1.14 (1.09, 1.19)	<0.001
Unadjusted RR	1.00 (reference)	1.08 (1.03, 1.13)	1.15 (1.10, 1.21)	1.22 (1.17, 1.28)	<0.001
Multivariable adjusted RR	1.00 (reference)	1.03 (0.98, 1.07)	1.06 (1.02, 1.11)	1.11 (1.06, 1.16)	<0.001
Case, n (%)/PY	3,520 (18.7)/113,505	3,762 (20.0)/112,205	3,977 (21.2)/111,092	4,184 (22.3)/110,608	
Incidence density	31.0	33.5	35.8	37.8	
Range of SIRI (median)	≤404 (320)	404–562 (480)	562–794 (662)	≥794 (1,023)	
NLR (n)	18,788	18,786	18,788	18,786	
Unadjusted HR	1.00 (reference)	1.10 (1.05, 1.15)	1.10 (1.05, 1.15)	1.15 (1.10, 1.20)	<0.001
Multivariable adjusted HR	1.00 (reference)	1.10 (1.05, 1.13)	1.08 (1.03, 1.13)	1.16 (1.11, 1.21)	<0.001
Unadjusted RR	1.00 (reference)	1.10 (1.04, 1.14)	1.09 (1.05, 1.14)	1.13 (1.08, 1.18)	<0.001
Multivariable adjusted RR	1.00 (reference)	1.08 (1.04, 1.13)	1.06 (1.02, 1.11)	1.13 (1.08, 1.18)	<0.001
Case, n (%)/PY	3,614 (19.2)/112,926	3,920 (20.9)/112,036	3,902 (20.8)/111,461	4,007 (21.3)/110,986	
Incidence density	32.0	35.0	35.0	36.1	
Range of NLR (median)	≤1.18 (1.01)	1.18–1.48 (1.33)	1.48–1.88 (1.65)	≥1.88 (2.25)	
MLR (n)	18,788	18,787	18,790	18,783	
Unadjusted HR	1.00 (reference)	1.03 (0.98, 1.08)	1.03 (0.98, 1.07)	1.04 (0.99, 1.09)	0.122
Multivariable adjusted HR	1.00 (reference)	1.06 (1.01, 1.10)	1.05 (1.01, 1.10)	1.10 (1.05, 1.15)	<0.001
Unadjusted RR	1.00 (reference)	1.02 (0.97, 1.07)	1.02 (0.97, 1.07)	1.02 (0.98, 1.07)	0.380
Multivariable adjusted RR	1.00 (reference)	1.03 (0.99, 1.08)	1.04 (1.00, 1.09)	1.07 (1.02, 1.12)	0.005
Case, n (%)/PY	3,827 (20.4)/112,537	3,862 (20.6)/111,505	3,885 (20.7)/112,075	3,869 (20.6)/111,292	
Incidence density	34.1	34.7	34.7	34.8	
Range of MLR (median)	≤0.145 (0.126)	0.145–0.179 (0.162)	0.179–0.220 (0.197)	≥0.220 (0.257)	
PLR (n)	18,787	18,787	18,787	18,787	
Unadjusted HR	1.00 (reference)	0.96 (0.92, 1.00)	0.91 (0.87, 0.96)	0.96 (0.92, 1.00)	0.028
Multivariable adjusted HR	1.00 (reference)	1.03 (0.98, 1.08)	1.03 (0.98, 1.08)	1.13 (1.08, 1.18)	<0.001
Unadjusted RR	1.00 (reference)	0.96 (0.92, 1.00)	0.91 (0.87, 0.95)	0.95 (0.91, 1.00)	0.001
Multivariable adjusted RR	1.00 (reference)	1.02 (0.98, 1.07)	1.02 (0.98, 1.07)	1.12 (1.07, 1.17)	<0.001
Case, n (%)/PY	4,025 (21.4)/111,409	3,873 (20.6)/112,019	3,694 (19.7)/112,127	3,851 (20.5)/111,855	
Incidence density	36.1	34.6	32.9	34.4	
Range of PLR (median)	≤91 (80)	91–109 (100)	109–130 (118)	≥130 (148)	

HR, hazard ratio; RR, relative risk; CI, confidence interval; SII, systemic immune-inflammation index; SIRI, systemic inflammatory response index; NLR, neutrophil-to-lymphocyte ratio; MLR, monocyte-to-lymphocyte ratio; PLR, platelet-to-lymphocyte ratio; PY, person years.

1 Adjusted for age, diabetes mellitus, physical activity, alcohol intake, smoking, body mass index, low-density lipoprotein cholesterol, triglycerides, homeostatic model assessment for insulin resistance, and systolic blood pressure.

**Table 4.** HRs, RR, and their 95% CIs for hypertension according to quartiles of SII, SIRI, NLR, MLR, and PLR in women

Variables^1^	Quartile 1	Quartile 2	Quartile 3	Quartile 4	p for trend
SII (n)	13,274	13,273	13,273	13,273	
Unadjusted HR	1.00 (reference)	1.09 (0.98, 1.22)	1.44 (1.29, 1.60)	1.72 (1.55, 1.91)	<0.001
Multivariable adjusted HR	1.00 (reference)	1.04 (0.93, 1.16)	1.23 (1.11, 1.37)	1.39 (1.25, 1.54)	<0.001
Unadjusted RR	1.00 (reference)	1.09 (0.98, 1.22)	1.43 (1.28, 1.59)	1.70 (1.54, 1.89)	<0.001
Multivariable adjusted RR	1.00 (reference)	1.04 (0.93, 1.16)	1.22 (1.10, 1.36)	1.36 (1.23, 1.51)	<0.001
Case, n (%)/PY	588 (4.4)/81,504	643 (4.8)/81,602	837 (6.3)/81,297	992 (7.5)/80,759	
Incidence density	7.2	7.9	10.3	12.3	
Range of SII (median)	≤292 (238)	292–390 (341)	390–523 (448)	≥523 (648)	
SIRI (n)	13,274	13,273	13,273	13,273	
Unadjusted HR	1.00 (reference)	1.09 (0.97, 1.21)	1.24 (1.12, 1.38)	1.41 (1.28, 1.56)	<0.001
Multivariable adjusted HR	1.00 (reference)	1.01 (0.91, 1.12)	1.14 (1.03, 1.27)	1.24 (1.12, 1.38)	<0.001
Unadjusted RR	1.00 (reference)	1.08 (0.97, 1.20)	1.23 (1.11, 1.37)	1.40 (1.26, 1.55)	<0.001
Multivariable adjusted RR	1.00 (reference)	1.03 (0.92, 1.14)	1.13 (1.02, 1.26)	1.22 (1.10, 1.35)	<0.001
Case, n (%)/PY	652 (4.9)/81,638	702 (5.3)/81,181	801 (6.0)/81,224	905 (6.8)/81,120	
Incidence density	8.0	8.7	9.9	11.2	
Range of SIRI (median)	≤365 (285)	365–519 (440)	519–743 (614)	≥743 (966)	
NLR (n)	13,274	13,273	13,274	13,272	
Unadjusted HR	1.00 (reference)	1.10 (0.99, 1.22)	1.29 (1.16, 1.42)	1.33 (1.20, 1.47)	<0.001
Multivariable adjusted HR	1.00 (reference)	1.12 (1.01, 1.25)	1.27 (1.15, 1.41)	1.34 (1.20, 1.48)	<0.001
Unadjusted RR	1.00 (reference)	1.09 (0.98, 1.22)	1.29 (1.16, 1.42)	1.30 (1.18, 1.44)	<0.001
Multivariable adjusted RR	1.00 (reference)	1.10 (0.99, 1.22)	1.25 (1.13, 1.39)	1.29 (1.16, 1.43)	<0.001
Case, n (%)/PY	655 (4.9)/81,436	715 (5.4)/81,297	841 (6.3)/81,317	849 (6.4)/81,113	
Incidence density	8.0	8.8	10.3	10.5	
Range of NLR (median)	≤1.25 (1.05)	1.25–1.59 (1.42)	1.59–2.04 (1.78)	≥2.04 (2.45)	
MLR (n)	13,274	13,273	13,275	13,271	
Unadjusted HR	1.00 (reference)	0.96 (0.86, 1.06)	1.06 (0.96, 1.18)	1.04 (0.94, 1.15)	0.166
Multivariable adjusted HR	1.00 (reference)	0.98 (0.88, 1.08)	1.11 (1.01, 1.23)	1.15 (1.04, 1.28)	0.001
Unadjusted RR	1.00 (reference)	0.96 (0.86, 1.06)	1.06 (0.96, 1.17)	1.04 (0.94, 1.15)	0.193
Multivariable adjusted RR	1.00 (reference)	0.98 (0.88, 1.08)	1.12 (1.01, 1.23)	1.14 (1.03, 1.26)	0.001
Case, n (%)/PY	753 (5.7)/81,207	720 (5.4)/81,144	802 (6.0)/81,415	785 (5.9)/81,397	
Incidence density	9.3	8.9	9.9	9.6	
Range of MLR (median)	≤0.137 (0.119)	0.137–0.170 (0.153)	0.170–0.212 (0.188)	≥0.212 (0.249)	
PLR (n)	13,274	13,273	13,273	13,273	
Unadjusted HR	1.00 (reference)	1.06 (0.95, 1.17)	1.11 (1.00, 1.23)	1.40 (1.27, 1.55)	<0.001
Multivariable adjusted HR	1.00 (reference)	1.02 (0.92, 1.14)	1.15 (1.04, 1.28)	1.37 (1.24, 1.51)	<0.001
Unadjusted RR	1.00 (reference)	1.05 (0.94, 1.17)	1.11 (1.00, 1.23)	1.39 (1.26, 1.53)	<0.001
Multivariable adjusted RR	1.00 (reference)	1.02 (0.92, 1.14)	1.15 (1.04, 1.28)	1.36 (1.23, 1.50)	<0.001
Case, n (%)/PY	675 (5.1)/81,433	707 (5.3)/81,317	746 (5.6)/81,423	932 (7.0)/80,989	
Incidence density	8.3	8.7	9.2	11.5	
Range of PLR (median)	≤106 (93)	106–127 (117)	127–154 (139)	≥154 (177)	

HR, hazard ratio; RR, relative risk; CI, confidence interval; SII, systemic immune-inflammation index; SIRI, systemic inflammatory response index; NLR, neutrophil-to-lymphocyte ratio; MLR, monocyte-to-lymphocyte ratio; PLR, platelet-to-lymphocyte ratio; PY, person years.

1 Adjusted for age, diabetes mellitus, physical activity, alcohol intake, smoking, body mass index, low-density lipoprotein cholesterol, triglycerides, homeostatic model assessment for insulin resistance, and systolic blood pressure.

**Table 5.** HRs, RR, and their 95% CIs for hypertension according to quartiles of hsCRP

Variables^1^	Quartile 1	Quartile 2	Quartile 3	Quartile 4	p for trend
All participants (n)	30,874	29,238	24,099	26,854	
Unadjusted HR	1.00 (reference)	1.66 (1.58, 1.74)	2.12 (2.02, 2.22)	2.50 (2.39, 2.62)	<0.001
Multivariable adjusted HR	1.00 (reference)	1.07 (1.02, 1.13)	1.10 (1.04, 1.16)	1.17 (1.11, 1.24)	<0.001
Unadjusted RR	1.00 (reference)	1.68 (1.60, 1.76)	2.12 (2.02, 2.23)	2.48 (2.37, 2.60)	<0.001
Multivariable adjusted RR	1.00 (reference)	1.08 (1.03, 1.14)	1.11 (1.05, 1.17)	1.17 (1.11, 1.24)	<0.001
Incidence case, n (%)/PY	2,590 (8.4)/191,899	4,088 (14.0)/180,387	4,171 (17.3)/145,694	5,359 (20.0)/160,053	
Incidence density	13.5	22.7	28.6	33.5	
Range of hsCRP (median)	≤0.02 (0.02)	0.03–0.04 (0.03)	0.05–0.08 (0.06)	≥0.09 (0.15)	
Men (n)	22,261	13,508	14,919	15,071	
Unadjusted HR	1.00 (reference)	1.26 (1.20, 1.33)	1.41 (1.34, 1.47)	1.58 (1.51, 1.65)	<0.001
Multivariable adjusted HR	1.00 (reference)	1.04 (0.99, 1.09)	1.06 (1.01, 1.12)	1.10 (1.05, 1.16)	<0.001
Unadjusted RR	1.00 (reference)	1.30 (1.23, 1.38)	1.49 (1.41, 1.58)	1.73 (1.64, 1.82)	<0.001
Multivariable adjusted RR	1.00 (reference)	1.07 (1.01, 1.15)	1.08 (1.02, 1.13)	1.15 (1.08, 1.22)	0.001
Incidence case, n (%)/PY	3,749 (16.9)/137,226	2,819 (20.9)/81,297	3,359 (22.7)/88,357	3,668 (24.9)/86,525	
Incidence density	27.3	34.7	38.0	42.4	
Range of hsCRP (median)	≤0.03 (0.02)	0.04–0.05 (0.04)	0.06–0.10 (0.07)	≥0.11 (0.18)	
Women (n)	18,785	7,309	8,986	10,822	
Unadjusted HR	1.00 (reference)	1.25 (1.10, 1.41)	1.66 (1.49, 1.85)	2.08 (1.89, 2.29)	<0.001
Multivariable adjusted HR	1.00 (reference)	1.01 (0.90, 1.15)	1.19 (1.07, 1.33)	1.18 (1.06, 1.32)	0.006
Unadjusted RR	1.00 (reference)	1.36 (1.23, 1.51)	1.76 (1.57, 1.97)	2.19 (1.98, 2.43)	<0.001
Multivariable adjusted RR	1.00 (reference)	1.08 (0.97, 1.20)	1.20 (1.06, 1.34)	1.22 (1.08, 1.37)	0.009
Incidence case, n (%)/PY	754 (4.0)/116,928	376 (5.1)/45,923	596 (6.6)/55,315	887 (8.2)/66,462	
Incidence density	6.5	8.2	10.8	13.3	
Range of hsCRP (median)	≤0.02 (0.02)	0.03–0.03 (0.03)	0.04–0.06 (0.05)	≥0.07 (0.13)	

HR, hazard ratio; RR, relative risk; CI, confidence interval; hsCRP, high-sensitivity C-reactive protein; PY, person years.

1 Adjusted for age, diabetes mellitus, physical activity, alcohol intake, smoking, body mass index, low-density lipoprotein cholesterol, triglyceride, homeostatic model assessment for insulin resistance, and systolic blood pressure.

## Supplementary materials

Supplementary Material 1. (a) Kaplan-Meier curve for hypertension free survival probability in SII quartile (All participants) (b) Kaplan-Meier curve for hypertension free survival probability in SIRI quartile (All participants) (c) Kaplan-Meier curve for hypertension free survival probability in NLR quartile (All participants) (d) Kaplan-Meier curve for hypertension free survival probability in MLR quartile (All participants) (e) Kaplan-Meier curve for hypertension free survival probability in PLR quartile (All participants)

Supplementary Material 2. (a) Kaplan-Meier curve for hypertension free survival probability in SII quartile (Men) (b) Kaplan-Meier curve for hypertension free survival probability in SIRI quartile (Men) (c) Kaplan-Meier curve for hypertension free survival probability in NLR quartile (Men) (d) Kaplan-Meier curve for hypertension free survival probability in MLR quartile (Men) (e) Kaplan-Meier curve for hypertension free survival probability in PLR quartile (Men)

Supplementary Material 3. (a) Kaplan-Meier curve for hypertension free survival probability in SII quartile (Women) (b) Kaplan-Meier curve for hypertension free survival probability in SIRI quartile (Women) (c) Kaplan-Meier curve for hypertension free survival probability in NLR quartile (Women) (d) Kaplan-Meier curve for hypertension free survival probability in MLR quartile (Women) (e) Kaplan-Meier curve for hypertension free survival probability in PLR quartile (Women)
