# Supplementary material for: Hematological inflammatory indices and their relationship to the risk of hypertension
Source: Epidemiol Health. 2026 Feb 4;48:e2026008. doi: 10.4178/epih.e2026008 (PMC13033440; doi:10.4178/epih.e2026008)
Supplement: Supplementary Material 1. — (a) Kaplan-Meier curve for hypertension free survival probability in SII quartile (All participants) (b) Kaplan-Meier curve for hypertension free survival probability in SIRI quartile (All participants) (c) Kaplan-Meier curve for hypertension free survival probability in NLR quartile (All participants) (d) Kaplan-Meier curve for hypertension free survival probability in MLR quartile (All participants) (e) Kaplan-Meier curve for hypertension free survival probability in PLR quartile (All participants) [file epih-48-e2026008-Supplementary-1.pptx]

## Slide 1
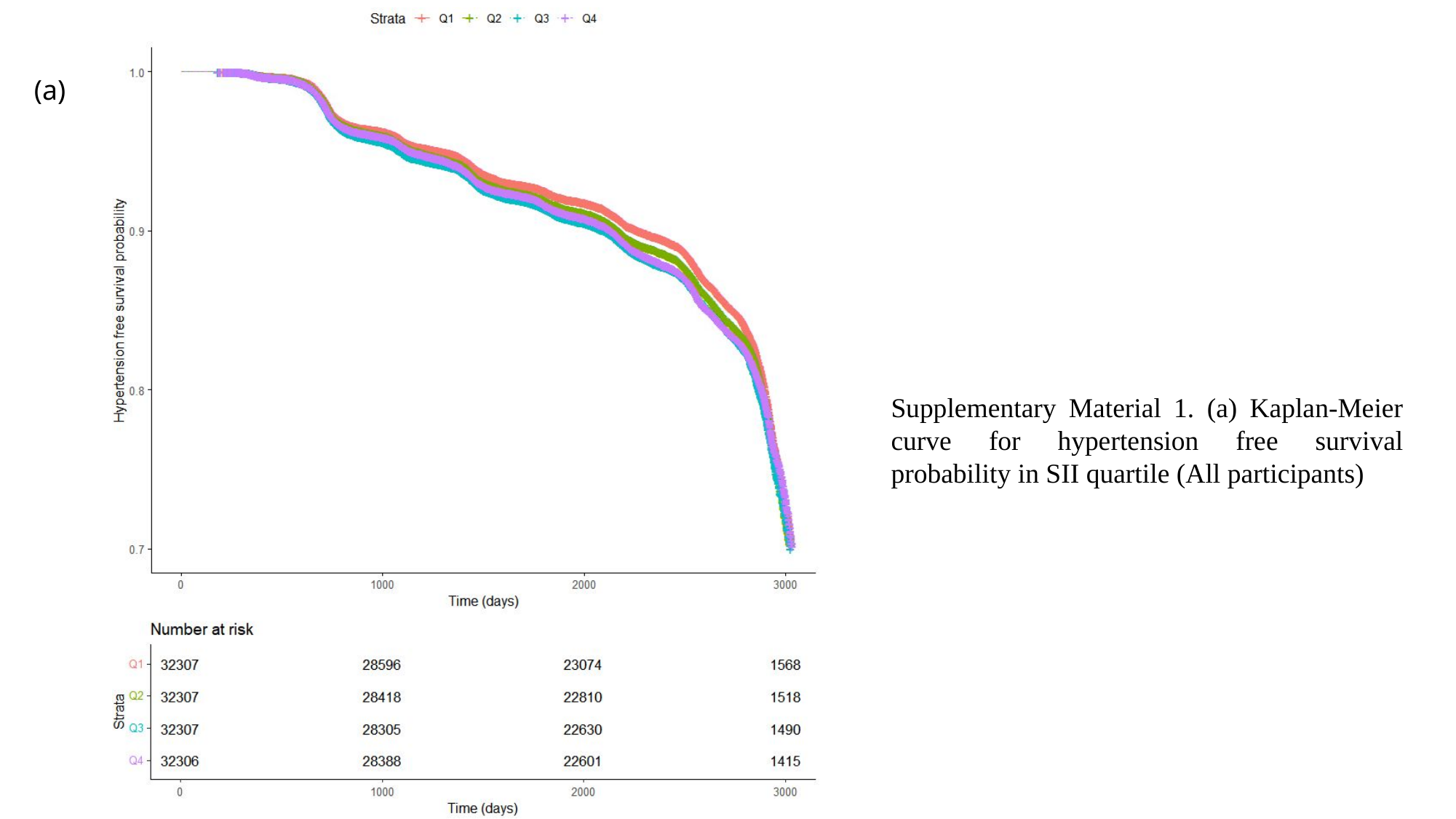

(a)
Supplementary Material 1. (a) Kaplan-Meier curve for hypertension free survival probability in SII quartile (All participants)

## Slide 2
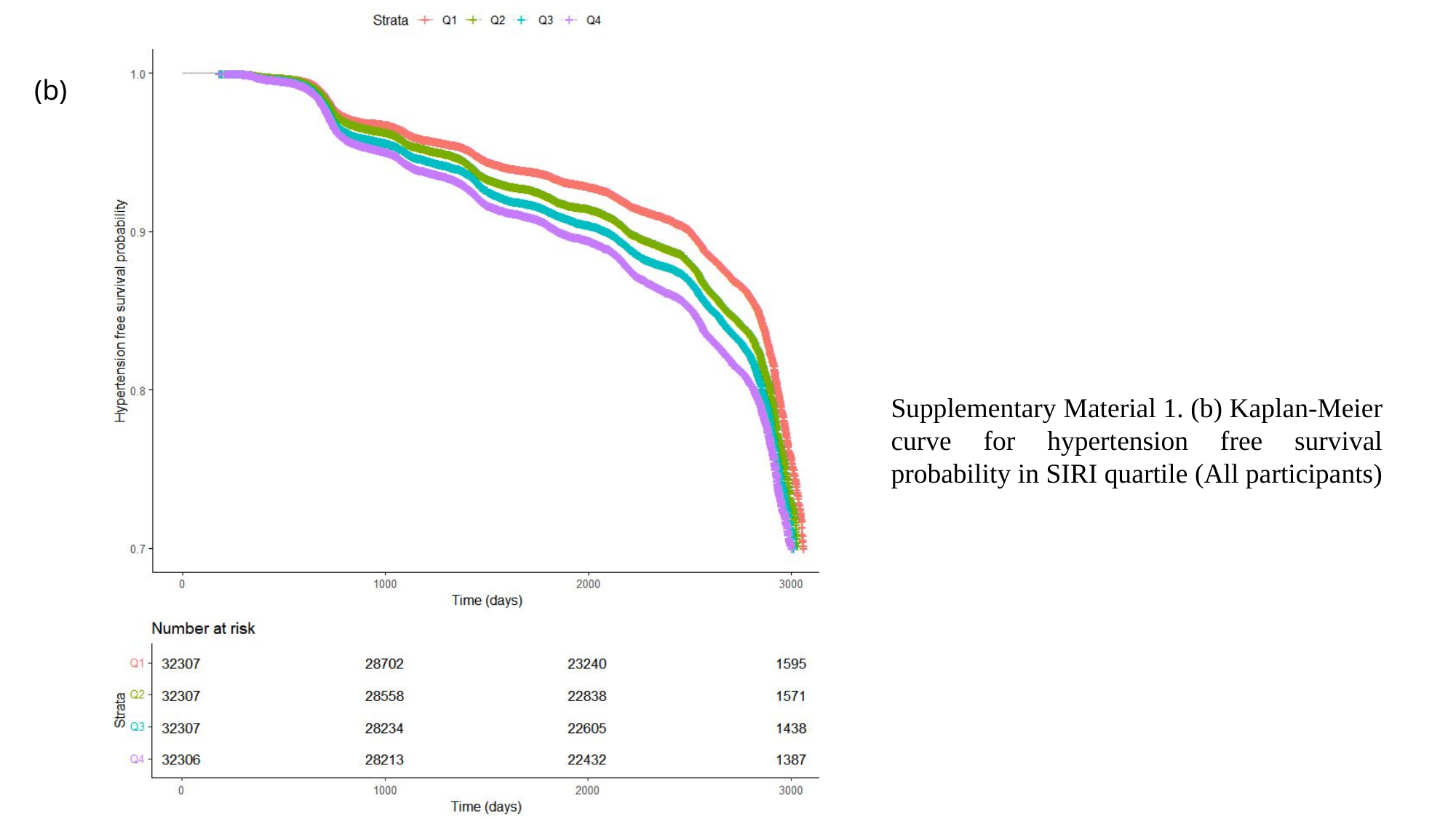

(b)
Supplementary Material 1. (b) Kaplan-Meier curve for hypertension free survival probability in SIRI quartile (All participants)

## Slide 3
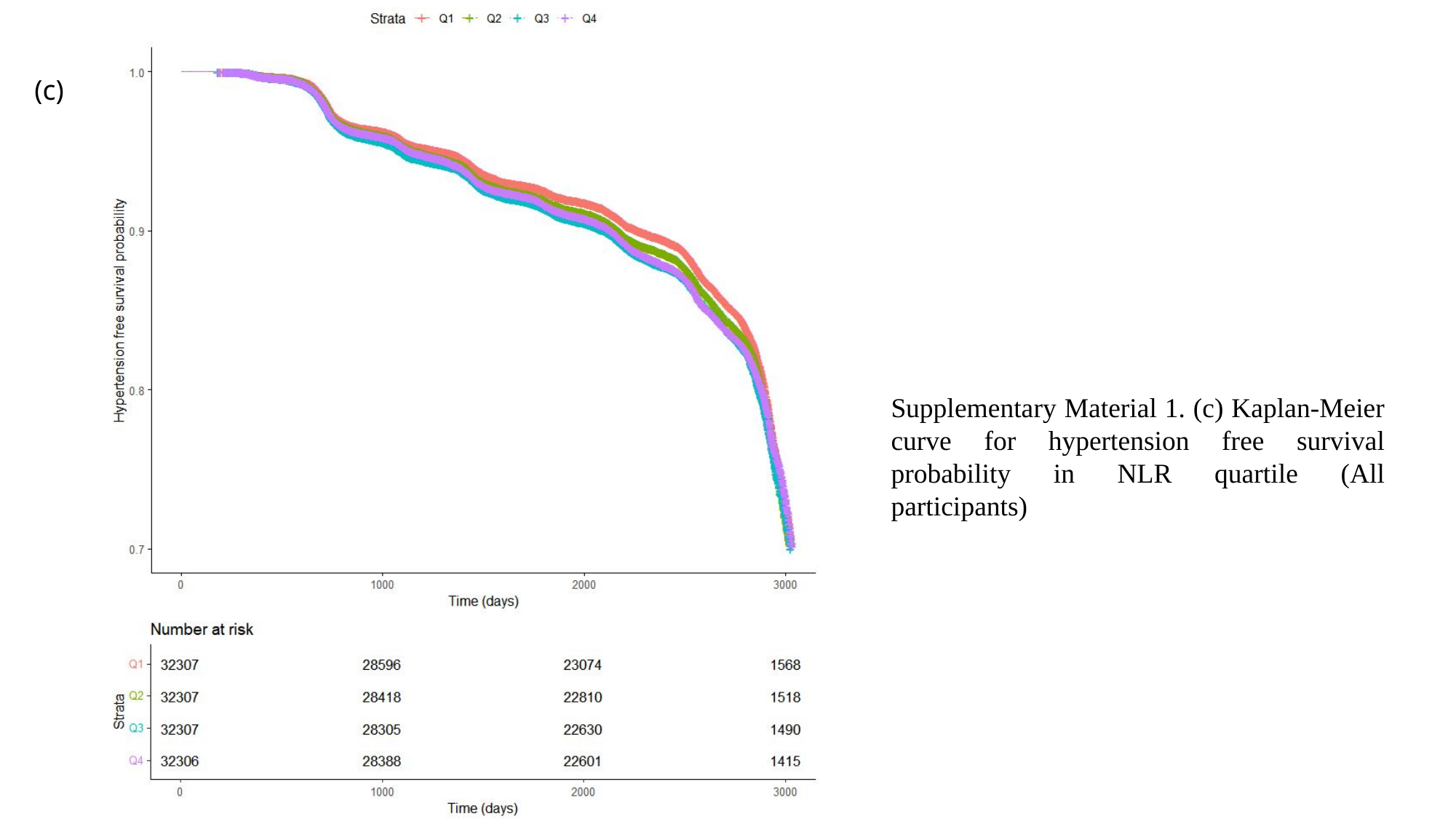

(c)
Supplementary Material 1. (c) Kaplan-Meier curve for hypertension free survival probability in NLR quartile (All participants)

## Slide 4
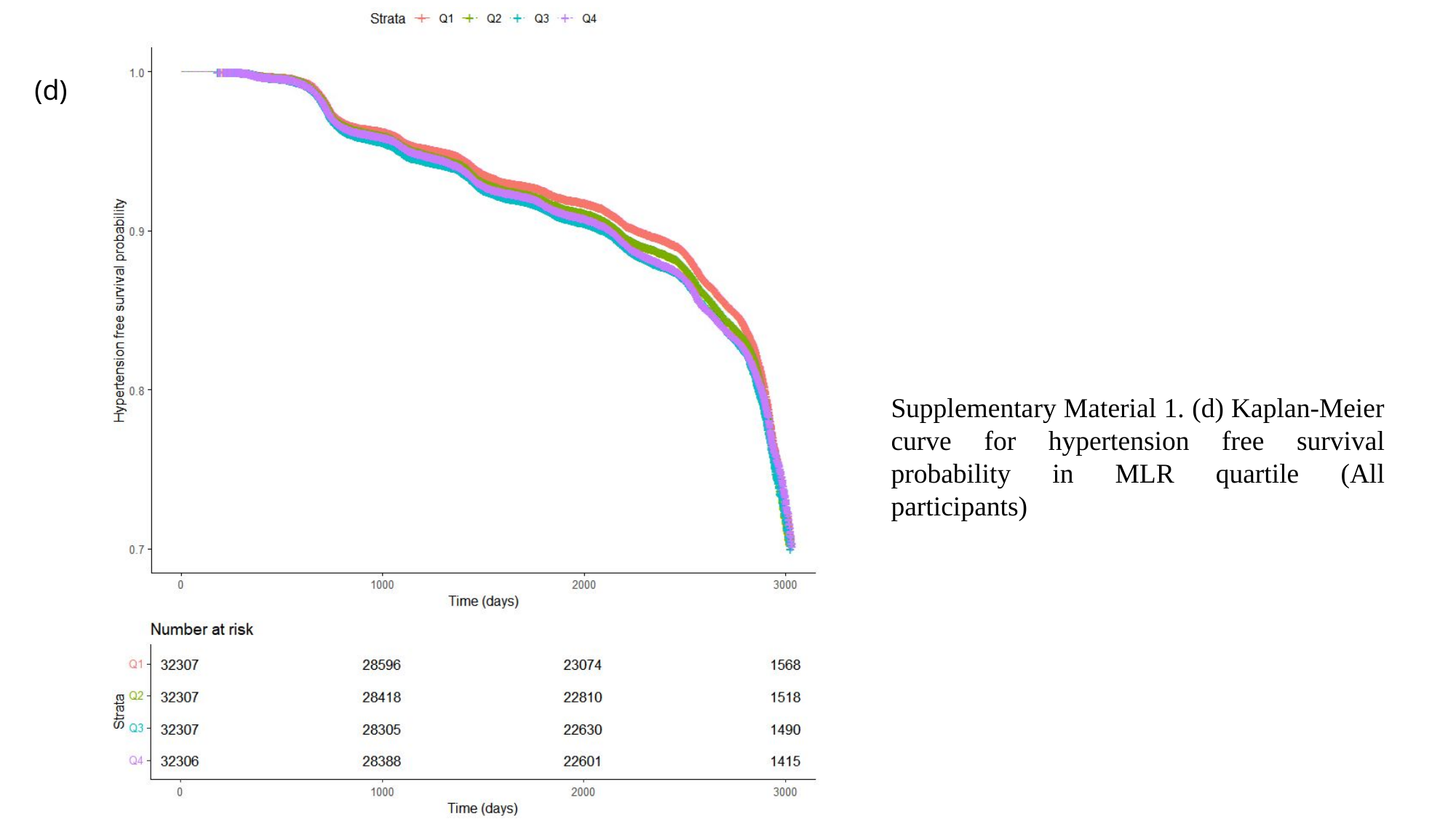

(d)
Supplementary Material 1. (d) Kaplan-Meier curve for hypertension free survival probability in MLR quartile (All participants)

## Slide 5
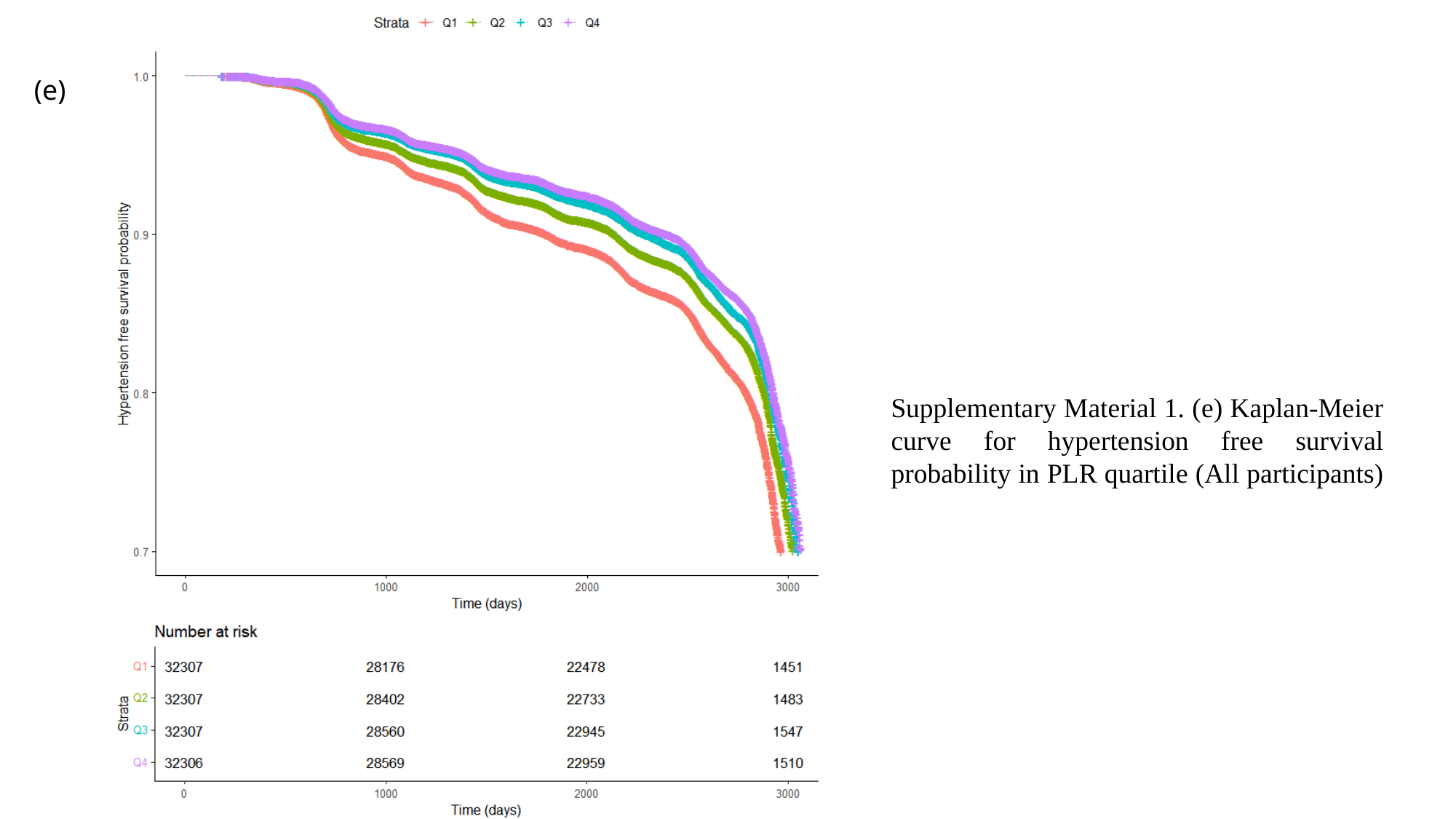

(e)
Supplementary Material 1. (e) Kaplan-Meier curve for hypertension free survival probability in PLR quartile (All participants)
