# Supplementary material for: Hematological inflammatory indices and their relationship to the risk of hypertension
Source: Epidemiol Health. 2026 Feb 4;48:e2026008. doi: 10.4178/epih.e2026008 (PMC13033440; doi:10.4178/epih.e2026008)
Supplement: Supplementary Material 3. — (a) Kaplan-Meier curve for hypertension free survival probability in SII quartile (Women) (b) Kaplan-Meier curve for hypertension free survival probability in SIRI quartile (Women) (c) Kaplan-Meier curve for hypertension free survival probability in NLR quartile (Women) (d) Kaplan-Meier curve for hypertension free survival probability in MLR quartile (Women) (e) Kaplan-Meier curve for hypertension free survival probability in PLR quartile (Women) [file epih-48-e2026008-Supplementary-3.pptx]

## Slide 1
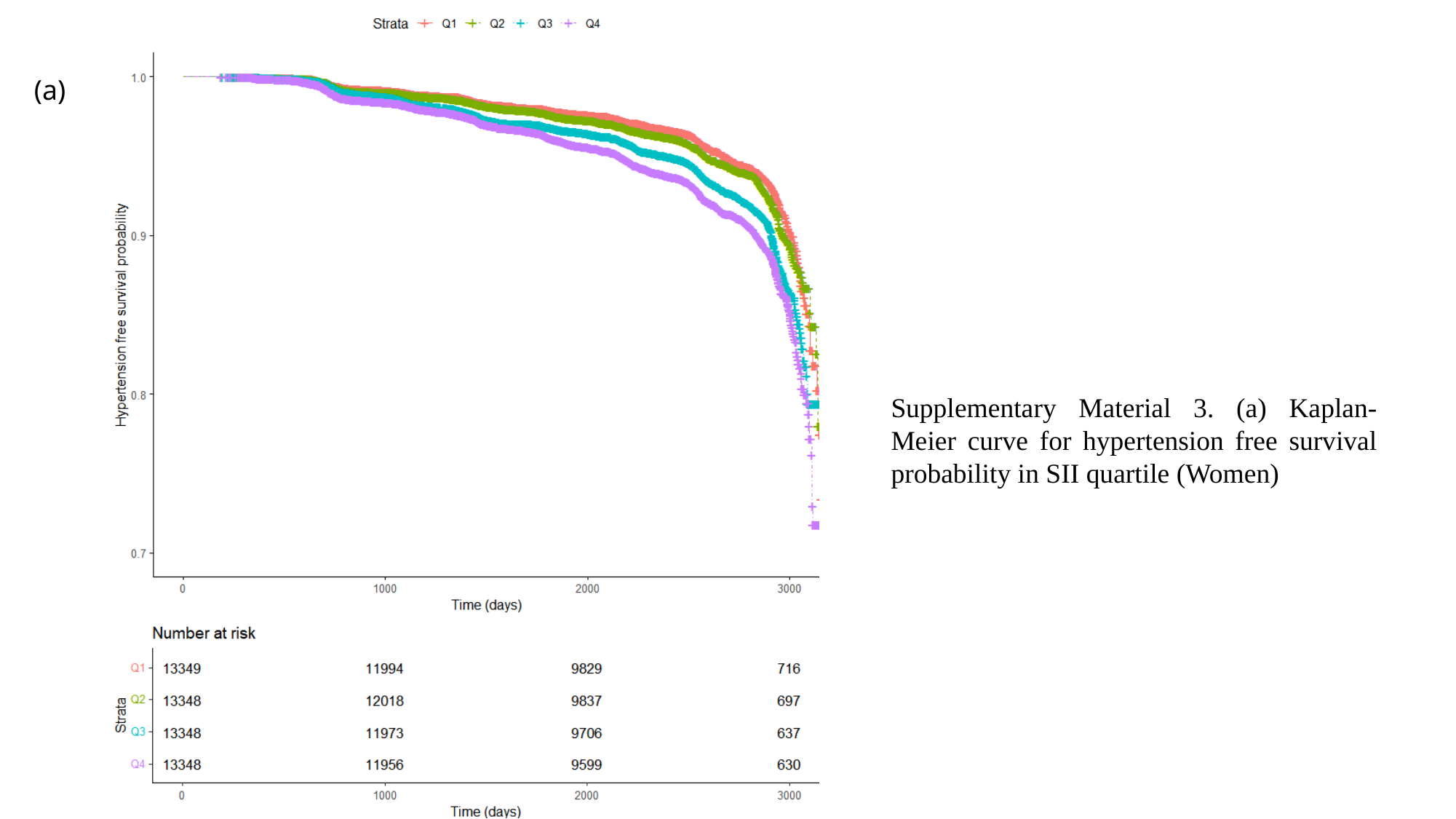

(a)
Supplementary Material 3. (a) Kaplan-Meier curve for hypertension free survival probability in SII quartile (Women)

## Slide 2
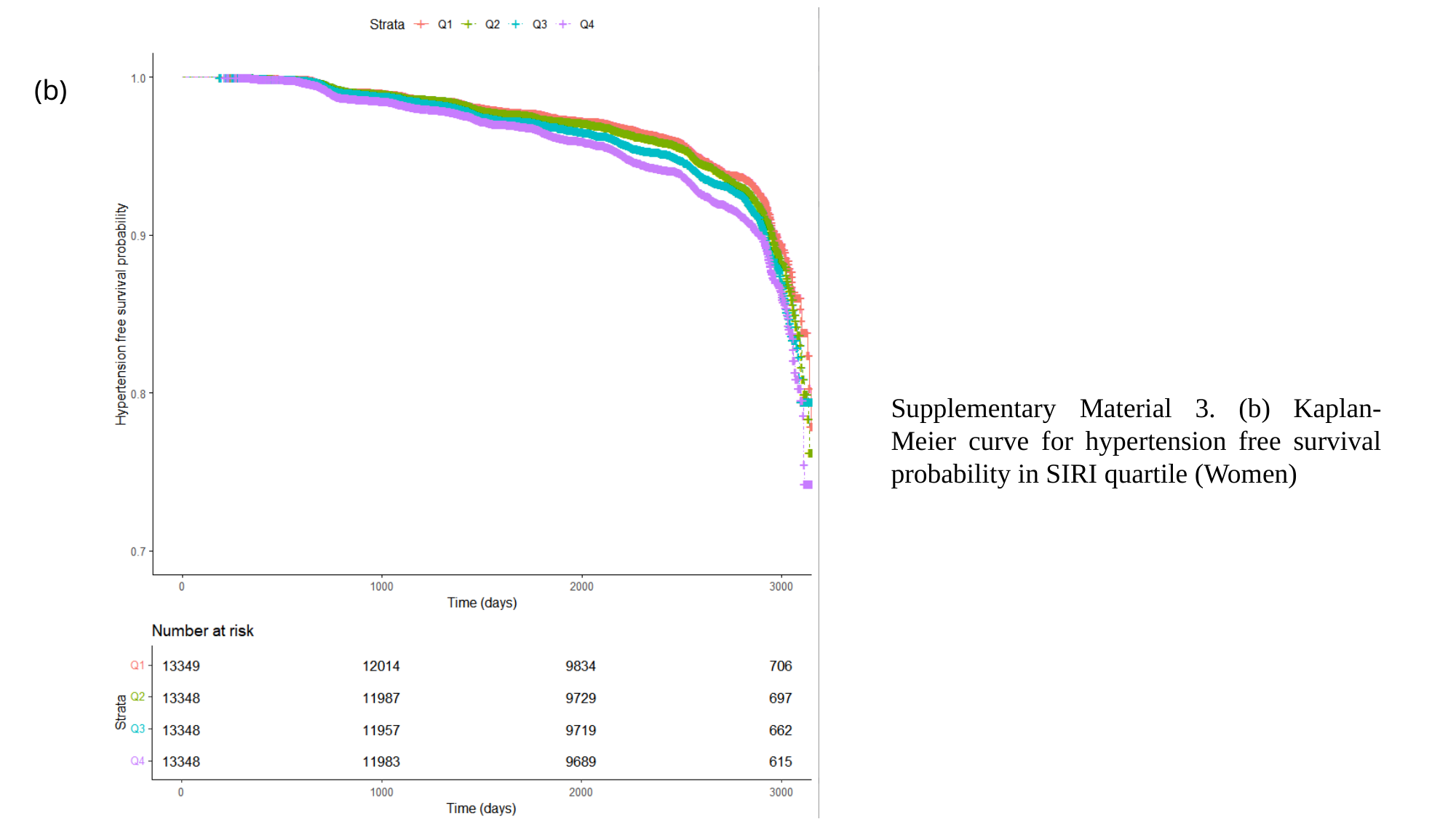

(b)
Supplementary Material 3. (b) Kaplan-Meier curve for hypertension free survival probability in SIRI quartile (Women)

## Slide 3
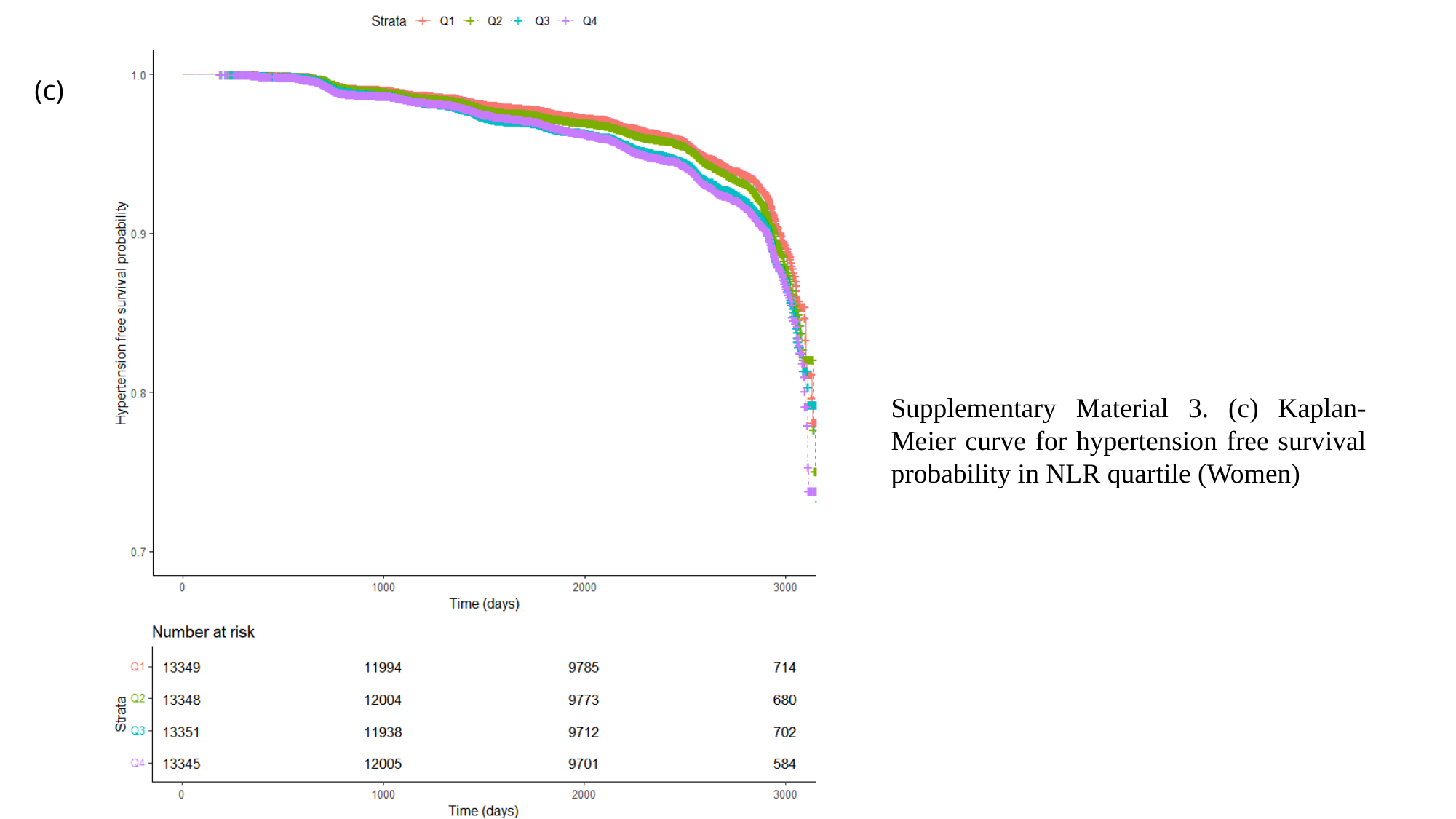

(c)
Supplementary Material 3. (c) Kaplan-Meier curve for hypertension free survival probability in NLR quartile (Women)

## Slide 4
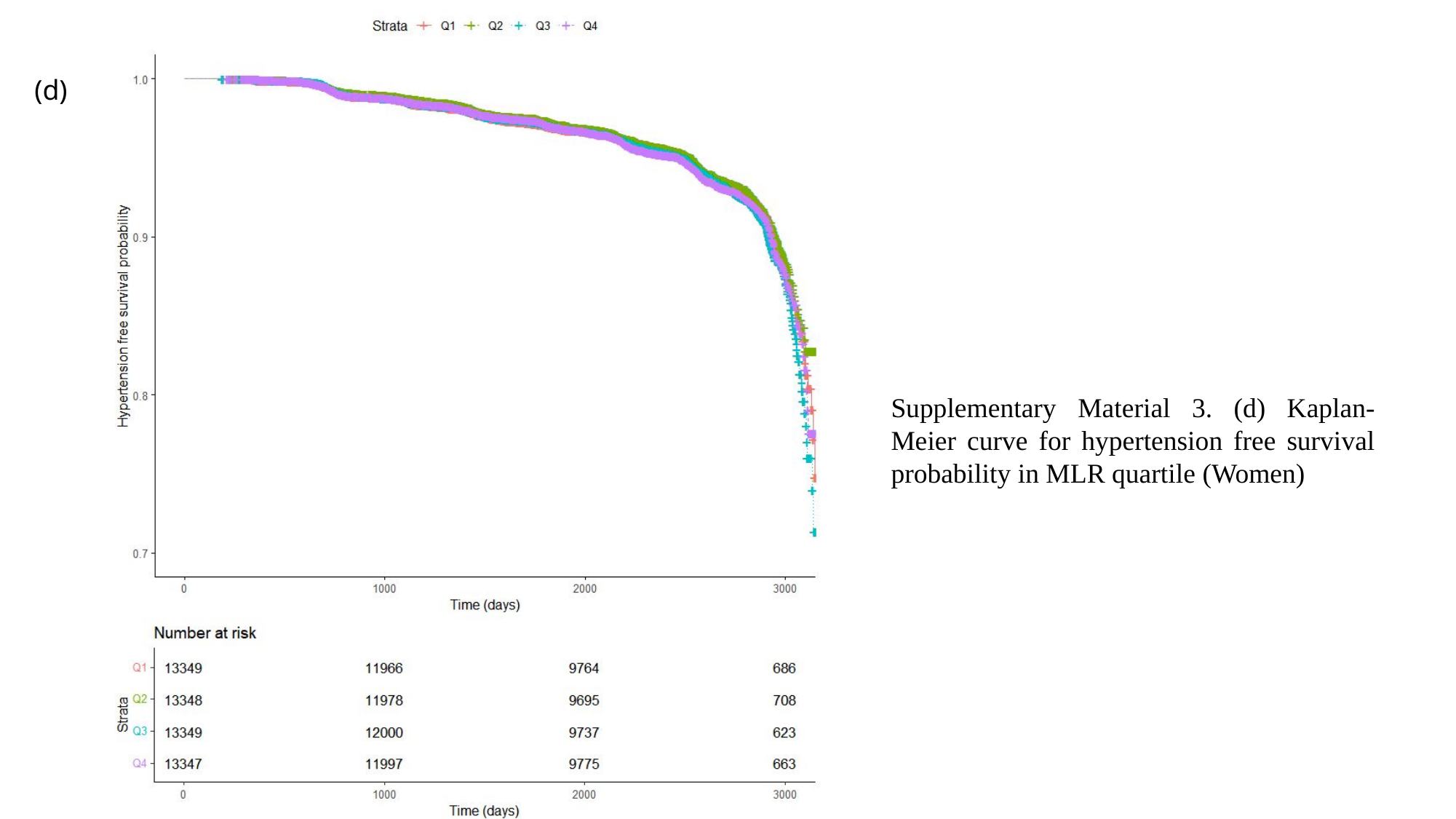

(d)
Supplementary Material 3. (d) Kaplan-Meier curve for hypertension free survival probability in MLR quartile (Women)

## Slide 5
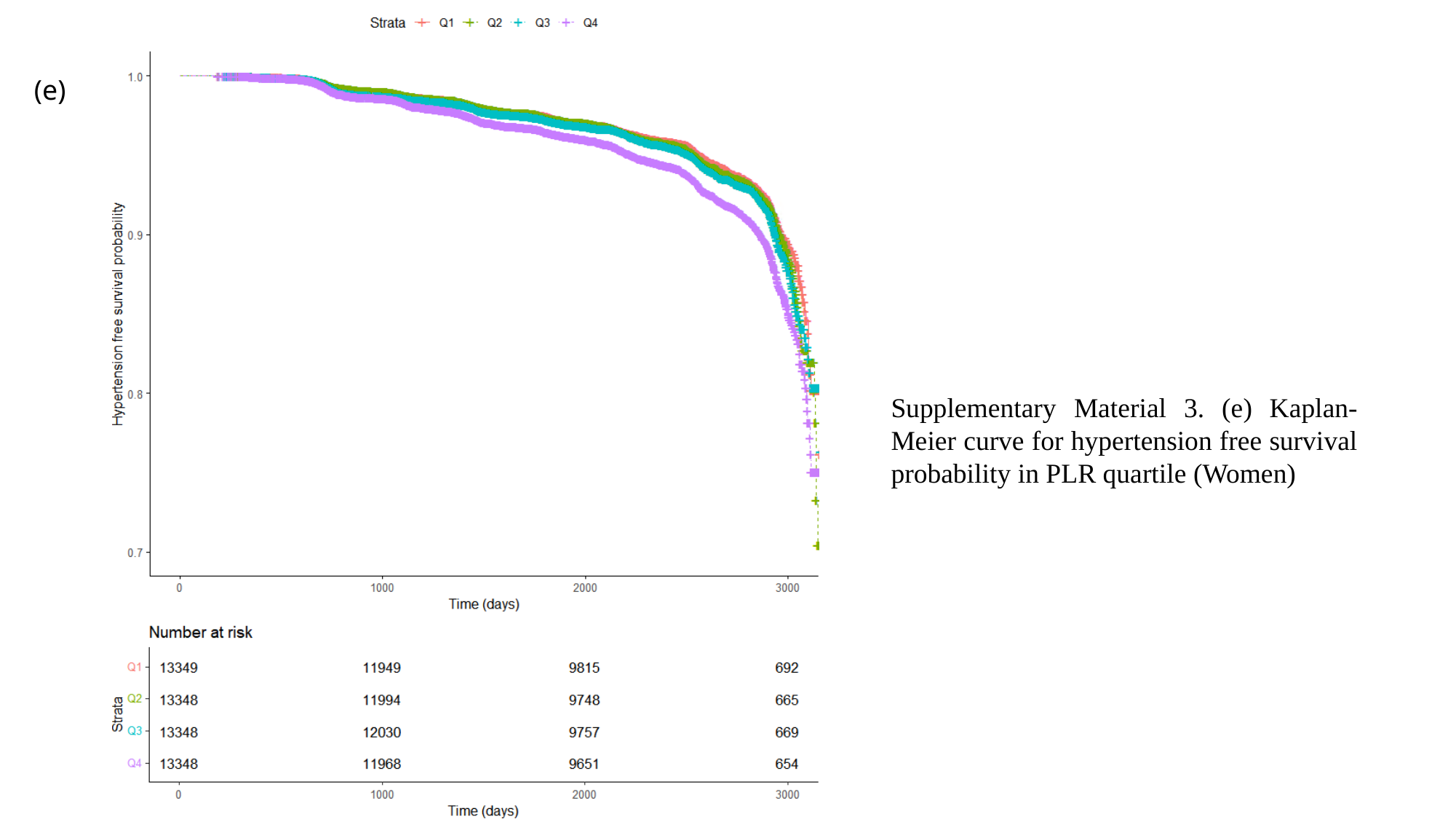

(e)
Supplementary Material 3. (e) Kaplan-Meier curve for hypertension free survival probability in PLR quartile (Women)
